# Sigma viruses from three species of *Drosophila* form a major new clade in the rhabdovirus phylogeny

**DOI:** 10.1098/rspb.2009.1472

**Published:** 2009-10-07

**Authors:** Ben Longdon, Darren J. Obbard, Francis M. Jiggins

**Affiliations:** 1 Institute of Evolutionary Biology, University of Edinburgh, Ashworth Laboratories, Kings Buildings, West Mains Road, Edinburgh EH9 3JT, UK; 2 Department of Genetics, University of Cambridge, Cambridge CB2 3EH, UK

**Keywords:** *Drosophila*, sigma virus, rhabdovirus, phylogeny, insect-virus

## Abstract

The sigma virus (DMelSV), which is a natural pathogen of *Drosophila melanogaster*, is the only *Drosophila*-specific rhabdovirus that has been described. We have discovered two new rhabdoviruses, *D. obscura* and *D. affinis*, which we have named DObsSV and DAffSV, respectively. We sequenced the complete genomes of DObsSV and DMelSV, and the L gene from DAffSV. Combining these data with sequences from a wide range of other rhabdoviruses, we found that the three sigma viruses form a distinct clade which is a sister group to the Dimarhabdovirus supergroup, and the high levels of divergence between these viruses suggest that they deserve to be recognized as a new genus. Furthermore, our analysis produced the most robustly supported phylogeny of the *Rhabdoviridae* to date, allowing us to reconstruct the major transitions that have occurred during the evolution of the family. Our data suggest that the bias towards research into plants and vertebrates means that much of the diversity of rhabdoviruses has been missed, and rhabdoviruses may be common pathogens of insects.

## 1. Introduction

Rhabdoviruses are single-stranded negative sense RNA viruses in the order Mononegavirales. The family is diverse and has a wide host range, infecting plants, invertebrates and vertebrates (ICTVdB 2006). Rhabdoviruses were originally classified as a family based on their shared bullet-shaped morphology and on serological evidence, but genome sequencing has since confirmed their shared ancestry (Fu 2005). The rhabdoviruses are divided into six genera. The genus *Lyssavirus* infects a range of mammals and includes the rabies virus. The genera *Cytorhabdovirus* and *Nucleorhabdovirus* are arthropod-vectored and infect plants, while the genus *Novirhabdovirus* infects various species of fish. Members of the genera *Vesiculovirus* and *Ephemerovirus* infect a wide range of animals including fishes, invertebrates and mammals, and together form the dimarhabdovirus super group (Bourhy *et al*. 2005). A large proportion of the known dimarhabdoviruses have been isolated from vertebrates and arthropods, which are thought to vector them.

The full diversity of the rhabdovirus family is unknown because of a strong sampling bias towards lineages of agronomic and medical importance (Fu 2005; Ammar *et al*. 2009). One area of neglect is the study of rhabdoviruses in arthropod hosts. As the majority of known dimarhabdoviruses, cytorhabdoviruses and nucleorhabdoviruses are arthropod-vectored (often insect-vectored), by studying arthropod-specific rhabdoviruses we may be able to understand how and why these viruses evolved traits such as virulence towards vertebrates.

The only arthropod-specific rhabdovirus that has been described to date is the sigma virus (DMelSV), which is a natural pathogen of *Drosophila melanogaster* (L'Heritier & Teissier 1937; Contamine & Gaumer 2008). Sigma has an unusual mode of transmission, in that it is only transmitted vertically (through both eggs and sperm), and does not move horizontally between hosts. It was initially placed in the *Rhabdoviridae* based on its bullet-shaped viral particles (Berkalof *et al*. 1965; Teninges 1968), and this has subsequently been confirmed using sequence data (Bjorklund *et al*. 1996). However, only about half of DMelSV's approximately 12.7 kb genome has previously been sequenced (Teninges *et al*. 1993), and the full sequence of the L gene—which encodes the RNA-dependant RNA polymerase (RDRP)—is unknown (Huszar & Imler 2008). This has hampered phylogenetic analyses of DMelSV because the L gene contains conserved domains that are useful in determining the evolutionary relationships between distantly related viruses (Poch *et al*. 1989, 1990; Bourhy *et al*. 2005). Previous phylogenies that have included DMelSV have been based on the less-conserved N gene, but many have lacked strong statistical support or only included a few closely related viruses. This may explain why the different studies have found conflicting results, either placing DMelSV as a sister group to the vesiculoviruses, or as an outgroup to the ephemeroviruses and vesiculoviruses (Bjorklund *et al*. 1996; Hogenhout *et al*. 2003; Fu 2005; Kuzmin *et al*. 2006).

It is possible that rhabdoviruses may be common pathogens in insect populations. Flies infected with DMelSV become paralysed or die on exposure to high concentrations of CO_2_, whereas uninfected flies recover, and similar symptoms occur when other rhabdoviruses are injected into mosquitoes or *Drosophila* (Rosen 1980; Shroyer & Rosen 1983). It has also been noted that aphids have reduced longevity after CO_2_ exposure following rhabdovirus injection (Sylvester & Richardson 1992). There have been reports of CO_2_ sensitivity occurring in at least 15 other species of *Drosophila* (Brun & Plus 1980) and in *Culex* mosquitoes (Shroyer & Rosen 1983), suggesting that rhabdoviruses may be common in insects. The most extensive of these studies looked at CO_2_ sensitivity in *D. affinis* and *D. athabasca*, and found that the sensitivity was caused by a vertically transmitted infectious agent (Williamson 1959, 1961). However, it is not known if this agent is a rhabdovirus, as other viruses (e.g. DXV which was isolated from cell culture) can also cause sensitivity to anoxia in *Drosophila* (Teninges *et al*. 1979).

In this study we have identified two new rhabdoviruses associated with CO_2_ sensitivity in *D. obscura* and *D. affinis*. To see where these new *Drosophila* rhabdoviruses are placed within the phylogeny, we sequenced the L gene from all viruses. In addition, we have completed the genome sequence of DMelSV and the new virus in *D. obscura*. The L gene of these viruses was combined with all the rhabdoviruses L gene sequences available from public databases to produce the most comprehensive phylogeny of the *Rhabdoviridae* published to date.

## 2. Material and methods

### (a) Identifying and sequencing viruses

*Drosophila affinis* were collected from Raleigh NC, USA and *D. obscura* were collected from Essex, UK in the summer/autumn of 2007. Flies were collected by netting from yeasted fruit baits, and isofemale lines were created by placing single females in a vial of *Drosophila* medium and allowing them to lay eggs. Offspring were then exposed to pure CO_2_ for 15 min at 12°C, then placed at room temperature and examined 30 min later. The lines where the flies were dead or paralysed were used for RNA extractions. CO_2_-sensitive lines were stabilized (Brun & Plus 1980) by selecting female offspring that transmitted the virus to 100 per cent of their offspring and maintained in the laboratory for over 15 generations. RNA was also extracted from two lines of *D. melanogaster* infected with the Hap23 and Ap30 isolates of DMelSV (Gay 1978; Carpenter *et al*. 2007). Ap30 is known to be genetically distinct from all the other DMelSV isolates that have been sequenced. Total RNA was extracted using Trizol reagent (Invitrogen Corp, San Diego, CA, USA) in a chloroform–isoproponal extraction. RNA was then reverse-transcribed with MMLV reverse transcriptase (Invitrogen Corp) using random hexamer primers.

The L gene of rhabdoviruses contains highly conserved domains (Poch *et al*. 1989, 1990), and is the most conserved gene in rhabdoviruses and other non-segmented negative sense RNA viruses (Fu 2005). This conservation is useful in designing polymerase chain reaction (PCR) primers which will work on a range of rhabdoviruses. Rhabdovirus L gene sequences were downloaded from GenBank and were aligned (as amino acids) using ClustalW. We manually designed degenerate primers that are conserved across most of the dimarhabdoviruses (electronic supplementary material, table S1). PCR reactions with all primer combinations were carried out using a touchdown PCR cycle. PCR products were treated with exonuclease 1 and shrimp alkaline phosphatase to remove unused PCR primers and dNTPs, and then sequenced directly using BigDye reagents (ABI, Carlsbad California, USA) on an ABI capillary sequencer. A sequence's similarity to rhabdoviruses was confirmed using a tBLASTN search of GenBank.

Once a small region of the L gene had been sequenced, 3′ RACE (rapid amplification of cDNA ends) was used to reach the 3′-end of the L gene mRNA. RNA was reverse-transcribed using superscript (Invitrogen Corp) and a T linker primer (5′- GATCGAT[17]VN -3′). Products were then purified using a PCR purification column kit (Qiagen Corp, MD, USA), and concentrated to a volume of 10–20 µl in a rotoevaporator. A PCR reaction (Long-Range PCR kit, Invitrogen Corp) was carried out using 2 µl of the cDNA using a T-linker primer and a gene-specific forward primer. In some cases a nested PCR was required on the first PCR (which was diluted 1:10 first). Products were then sequenced by primer walking and sequences were assembled using Sequencher (v. 4.5/4.8; Gene Codes Corp).

To obtain the remainder of the L gene and to attempt to obtain the rest of the genome, 3′-RACE was carried out on the viral genome itself. A polyA tail was added to the 3′-end of the virus using polyA polymerase (PAP). Approximately 5 µg of total RNA, 4 units (0.8 µl) PAP (New England Biolabs), 2 µl 10× PAP buffer, 2 µl rATP (10 mM) (Promega Corporation) and RNase-free water to 20 µl was incubated at 37°C for 40 min. The RNA was then purified using a spin column kit (Zymo clean, Cambridge Biosciences, UK). The eluted RNA was then reverse-transcribed using superscript (Invitrogen Corp) and a T linker primer. A PCR reaction was carried out using 2 µl of the cDNA using a T-linker and a gene-specific primer (Long Range PCR kit, Invitrogen Corp). In some cases a nested PCR was required on the first PCR (which was diluted 1:10 first). Products were then sequenced by primer walking using the methods described above. Although most of the 3′-end of the DMelSV genome has already been sequenced, the 3′ leader sequence is unknown. Therefore, we also used this approach to acquire the DMelSV leader sequence.

To obtain the 5′ genomic trailer sequence, and to determine the N gene transcription initiation site 5′-RACE was used. For the 5′-RACE on the viral genome, a gene-specific primer was used for a reverse transcription reaction using superscript RT (Invitrogen Corp), whereas for the 5′-RACE on mRNA sequences a T-linker primer was used. Two 5′-RACE methods were used. In the first 1 µl of BSA (20×) and 1 µl of Manganese (20×) were added to the reverse transcription reaction. Twenty microlitres of the cDNA was then incubated overnight at 16°C with 5 µl buffer 2 (New England Biolabs), 6 µl dNTPs (2 mM), 1 µl Klenow enzyme (5000 U µl^−1^) (New England Biolabs), 1 µl (50 µM) TS-short primer (5′-GGTCTGGAGCTAGTGTTGTGGG-3′) and 17 µl water. This was then purified in a spin column PCR purification kit (Qiagen Corp), and was used with a gene-specific primer and the TS short primer for PCR amplification. In the second method the cDNA was first purified using a spin column purification kit (Qiagen Corp). A polyA tail was added to the cDNA by incubating 21.5 µl of the purified cDNA with 1 µl of terminal transferase (30 U µl^−1^) (Promega Corp), 6 µl 5× terminal transferase buffer and 1.5 µl of dATP (2 mM) at 37°C for 40 min, then at 70°C for 10 min. This was then used for PCRs with a gene-specific primer and a T-linker primer. In both methods nested PCRs on the first round of PCRs was required, after diluting the samples 1:10.

Once the initial sequences from the RACE were obtained, new primers were designed along the length of the gene. These were used for PCR reactions on random-hexamer reverse-transcribed cDNA, which were then sequenced in both directions (see above) to obtain high-quality sequence data. The GenBank accession numbers for our new sequences are GQ375258 (DMelSV-HAP23), AM689309 (DMelSV-Ap30), GQ410979 (DObsSV) and GQ410980 (DAffSV).

### (b) Phylogenetic analysis

To infer the phylogeny, we obtained all the available full-length L gene sequences from GenBank. L gene coding sequences and the three sigma virus L gene sequences were aligned as translated amino acid sequences using ClustalW. As some of the sequences are highly divergent, we employed three different approaches for aligning the sequences to ensure our results were robust and not sensitive to the alignment used. First, we aligned the full-length L gene sequences from all of the viruses, then the most conserved region of the L gene from all of the viruses (corresponding to nucleotides 1284–3862 of rabies virus L gene coding region, GenBank accession NC_001542), and finally the full L gene sequences of the dimarhabdoviruses (with three lyssaviruses as an outgroup). The conserved region and the alignments of the dimarhabdoviruses alignments are likely to be the most robust, as they do not include very different sequences that are hard to align. Human parainfluenza virus 1 was also included in the first two of these alignments as an outgroup to root the tree.

The phylogeny of these sequences was reconstructed using both a Bayesian and a maximum-likelihood approach. Bayesian posterior support values are less conservative than maximum-likelihood bootstrap support, and so both the values can be used as an upper and lower support for nodes (Douady *et al*. 2003). In addition to nucleotide models, we ran Bayesian analysis with amino acid sequences and models of protein evolution. In total we carried out nine analyses, using three different methods of inference and three different sequence alignments.

Phylogenies were created from the amino acid alignments translated back into nucleotides. For the maximum-likelihood trees, ModelTest (v. 3.7) (Posada & Crandall 1998) was used to estimate the model of sequence evolution and the analysis was run in PAUP (v. 4.0b10) (Swofford 1993). A parsimony tree created from tree bisection and reconnection with a heuristic search was used as a starting tree for the maximum-likelihood analysis. A general time-reversible model with a gamma distribution of rate variation and proportion of invariable sites was used. The maximum-likelihood analysis used a heuristic search with a nearest neighbour interchange algorithm. The substitution rate parameters, shape of the gamma distribution and proportion of invariable sites used were those estimated by ModelTest. Support for the nodes was calculated by bootstrapping and trees were drawn using FigTree (v. 1.2; http://tree.bio.ed.ac.uk/software/figtree/).

Bayesian trees were created using the MrBayes program (v. 3.1.2) (Huelsenbeck & Ronquist 2001). A general time-reversible model was used with a gamma distribution and a proportion of invariable sites, with parameters estimated from the data during the analysis. As there is likely to be a considerable amount of noise from third codon positions between these divergent sequences, a site-specific rate model was used allowing each codon position to have its own rate. Two runs of four chains were run for 2 000 000 MCMC generations (20 000 000 for the conserved region tree), with trees being sampled every 100 generations.

In addition, Bayesian amino acid trees were created using the MrBayes programme (v. 3.1.2) (Huelsenbeck & Ronquist 2001). A fixed rate model of protein evolution was assumed, and the phylogeny was reconstructed using a model jumping method. This allows switching between different models of amino acid substitution during the MCMC process, and all the models contribute to the final result and are weighted according to their posterior probability. A gamma distribution of rate variation among sites was used, with the shape estimated from the data. Two runs of four chains were run for 5 000 000 MCMC generations (1 000 000 for the dimarhabdovirus alignment tree), with trees being sampled every 100 generations.

The average s.d. of split frequencies between the two runs approaching zero, and the log-likelihood values of the cold chain becoming stable, were used to assess when to stop the run. The first 25 per cent of the trees were discarded to ensure that the chains had reached stationarity, and a consensus tree was created from the remaining trees. Figures were created using FigTree (v. 1.2) (http://tree.bio.ed.ac.uk/software/figtree/).

To compare the topology of the sigma virus phylogeny with the *Drosophila* phylogeny, we reconstructed the maximum-likelihood phylogeny of Dimarhabdoviruses using the full-length L gene alignment under the constraint that the sigma virus phylogeny follows that of the hosts (i.e. *D. affinis* and *D. obscura* form a monophyletic group). We then tested whether the likelihood of the constrained tree was significantly less than the unconstrained tree using a Shimodaira–Hasegawa test (SH test) in PAUP using the maximum-likelihood dimarhabodvirus trees (Shimodaira & Hasegawa 1999).

Accession numbers for the sequences used in the phylogenetic analysis are available as supplementary materials.

## 3. Results

### (a) Identification of two new *Drosophila* sigma viruses

In samples from wild populations, we detected one line of *D. affinis* from Raleigh NC, USA and two lines of *D. obscura* from Essex, UK that were paralysed or died after exposure to CO_2_. To test whether these lines were infected with a rhabdovirus, we created cDNA from the flies and attempted to amplify a region of the RDRP gene using PCR primers designed in conserved sequences. All the CO_2_-sensitive lines produced a PCR product, which was sequenced and confirmed to be rhabdovirus-like by BLAST searches. These viruses were tentatively named as *D. affinis* sigma virus (DAffSV) and *D. obscura* sigma virus (DObsSV).

### (b) Genome sequences

We next attempted to sequence the genomes of these newly discovered viruses and the *D. melanogaster* sigma virus (DMelSV). Our strategy was to first use the short sequences produced with the conserved primers as the basis for 3′-RACE on both the L gene mRNA and the negative sense genome, and then to sequence the trailer sequence using 5′-RACE. This allowed us to completely sequence the genome of one DMelSV isolate, and to sequence all of the genome except the short 3′ leader and 5′ trailer sequences from a second DMelSV isolate. We sequenced the whole genome except the short 5′ trailer sequence for one DObsSV isolate. We also sequenced the entire L gene of DAffSV, but were unable to retrieve the remainder of the genome, possibly owing to a poly-A region causing mispriming of the T-linker primer.

### (c) DObsSV genome

**Figure 1.**
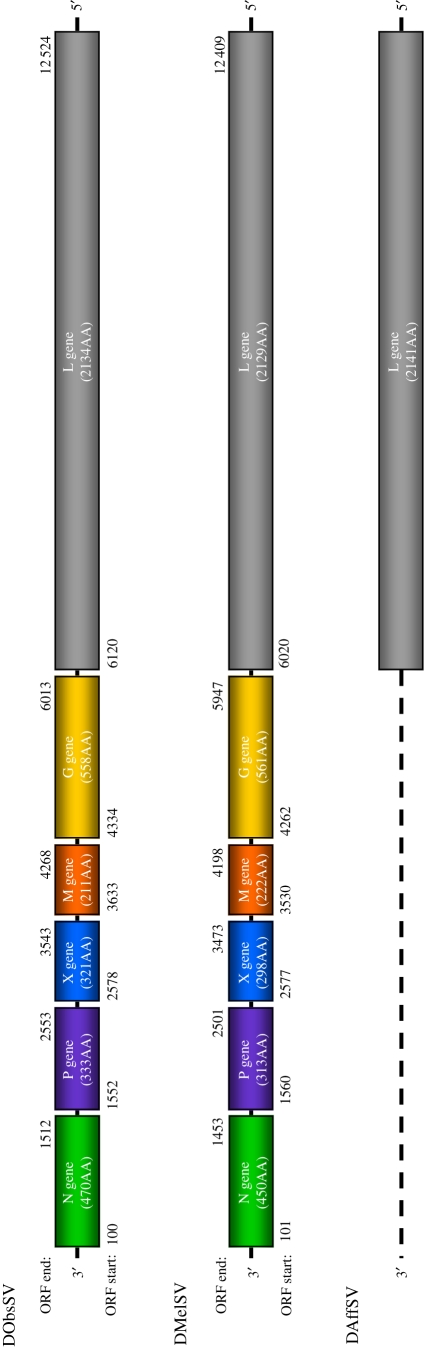
The sigma virus genomes. Numbers below show the position of the start codon, numbers above represent the stop codon. Dotted lines represent parts of the genome we were unable to sequence. In DMelSV the mRNA transcripts for the M and the G genes overlap by 33 bps, but the open reading frames do not overlap. Note that the X gene has also been referred to as gene 3 in some literature.

The genome of DObsSV (excluding the 5′ trailer) is 12 676 bp long. There are six open reading frames which, based on their predicted protein sequence and gene order, appear to be homologous to the N-P-X-M-G-L genes in DMelSV (figure 1). The N, P and X genes are in reading frame one, the M and the L genes are in frame two and the G gene is in frame three.

To annotate the coding sequence, we have assumed that each open reading frame starts at the first AUG occurring after the previous transcription termination sequence, and continues to the first stop codon. These coding regions make up 96 per cent of the genome, with the L gene covering 51 per cent of the total genome (figure 1). A tBLASTn search of the NCBI nucleotide collection using the predicted protein sequences of these genes returned significant alignments (blast alignment scores over 80) with homologous genes from other rhabdoviruses for the N, G and L genes. The M gene (which is thought to encode the matrix protein in DMelSV) had a weakly significant alignment to Flanders virus M gene and no significant alignments were found with the P or X genes, which are the least conserved genes in the genome.

To predict the structure and function of the P and X, we used PHYRE (Kelley & Sternberg 2009), which compares the query sequence with proteins of known structure and function, Interproscan, which searches for protein signatures in the InterPro database (Zdobnov & Apweiler 2001), and SignalP, which predicts signal peptides (Bendtsen *et al*. 2004). The X gene contains a signal peptide (SignalP: *p* = 0.98), but no predicted transmembrane regions, and has regions that are similar to viron RNA polymerases (PHYRE: 90% estimated precision), as has been reported for its homologue in DMelSV (Landesdevauchelle *et al*. 1995). However, it also shares similarities to topoisomerases, signal proteins and toxin molecules. We identified structures in the P gene as a viral RNA polymerase (PHYRE: 85% estimated precision).

In the non-coding regions, the motif 3′-GGUACUUUUUUU-5′ is found after all of the first five open reading frames in the genome. Based on its homology to other rhabdoviruses it is likely that it acts as the transcription termination sequence, and the seven U residues trigger polyadenylation of mRNAs (Huszar & Imler 2008). At the 3′ end of the genome there is a 3′ leader sequence of 99 bases before the first ATG. 5′ RACE on viral mRNAs failed, possibly owing to the T-linker primer annealing to polyA regions in the positive sense genomic strand, meaning we were unable to confirm the transcription initiation sequence.

### (d) DMelSV genome

The genome of DMelSV is 12 625 bp long, the first five genes of which have been published previously (Teninges *et al*. 1993; Bras *et al*. 1994; Landesdevauchelle *et al*. 1995). The newly sequenced L gene open reading frame is 6389 bases long and compromises 51 per cent of the total genome (figure 1). By using RACE to confirm the sequence at the start of the N gene and end of the L gene, we were able to annotate the 54 bp 3′ leader sequence and 180 bp trailer sequence. In DMelSV, the transcription initiation site is 3′-GUUGUNG-5′ (Teninges *et al*. 1993) for all the genes bar the N, where it is 3′-UUGUUG-5′. The transcription initiation site occurs shortly after the previous transcription termination signal, with the exception of the M/G gene junction where the M gene and G gene mRNAs overlap by 33 bases (Teninges *et al*. 1993). The protein-coding regions, however, do not overlap. In addition we found the G gene to be 21 amino acids longer than described in its original GenBank annotation owing to what was possibly a sequencing error causing a false stop codon (accession number X91062) (Landesdevauchelle *et al*. 1995). In sequencing the 5′ trailer region and comparing this with the L gene mRNA we found the same transcription termination sequence (3′-GUACUUUUUUU-5′) as previously reported (Teninges *et al*. 1993), at the end of the L gene.

### (e) DAffSV L gene

We sequenced the entire L gene and the 5′ trailer of DAffSV (figure 1). The predicted protein-coding sequence of the L gene has significant tBLASTN alignments to other Rhabdovirus L genes. The transcription termination sequence is the same as DMelSV (3′-GAUCUUUUUUU-5′) based on comparing where the L gene mRNA terminates (sequenced by 3′ RACE on the mRNA) with the genome sequence (sequenced by 5′ RACE on genomic RNA).

### (f) Sequence conservation

The amount of protein sequence divergence between the three sigma viruses is very similar, suggesting that they all diverged at a similar time (electronic supplementary material, table S2). However, the different genes in the genome have very different levels of amino acid sequence conservation (electronic supplementary material, table S2), with the L gene being the most conserved, and the P, X and M genes the least conserved.

There is a high level of amino acid sequence divergence between the three *Drosophila* sigma viruses (electronic supplementary material, table S3). Comparing the amino acid sequences of the L genes of the sigma viruses with those from related clades, we see that DMelSV, DObsSV and DAffSV share only a slightly higher sequence identity to one another than they do to viruses in a range of different rhabdovirus genera (electronic supplementary material, table S3). Furthermore, the amino acid sequence divergence between the three sigma viruses is only slightly less than that seen when rhabdoviruses in different genera are compared, and is similar to the maximum divergence seen between rhabdoviruses in the same genus.

### (g) Phylogeny of the *Rhabdoviridae*

**Figure 2.**
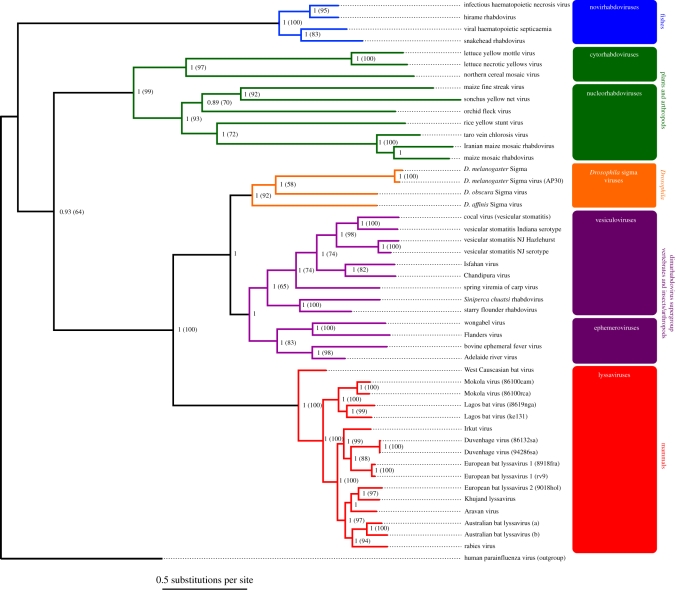
Phylogeny of the *Rhabdviridae* from full-length nucleotide sequences of the L gene. The tree was reconstructed using the Bayesian method, node labels are posterior supports and labels in brackets are maximum-likelihood bootstrap supports (500 replicates). The tree is rooted with human parainfluenza virus 1. The following viruses are unassigned rhabdoviruses; Flanders virus, Iranian maize mosaic virus, starry flounder rhabdovirus, *Siniperca chuatsi* rhabdovirus, wongabel virus, taro vein chlorosis virus, maize fine streak virus, lettuce yellow mottle virus, orchid fleck virus, but have been included in the different genera for illustrative purposes, based on their position in the phylogeny.

**Figure 3.**
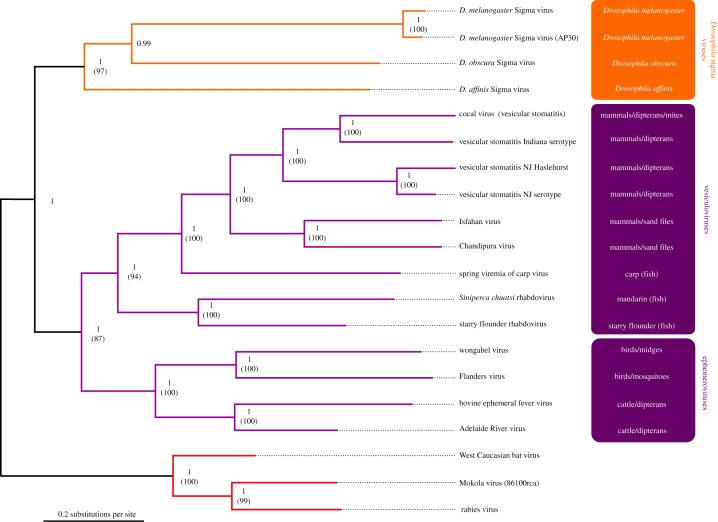
Bayesian nucleotide tree using full-length sequence alignments for the dimarhabdovirus and sigma virus clades. Node labels are posterior supports, labels in brackets are bootstrap supports taken from the maximum likelihood tree with 1000 bootstrap replicates. The tree is rooted with three lyssaviruses (West Caucasian bat virus, Mokola virus 86101RCA and rabies virus).

The phylogeny of the *Rhabdoviridae* was reconstructed from both full-length and conserved regions of L gene sequence. The three sigma viruses form a well-supported monophyletic group that is distinct from the other rhabdoviruses (figures 2 and 3). As was seen in the analysis of sequence identity, the divergence between the viruses is substantial, and similar to that between the most divergent members of some genera. Therefore, these viruses constitute a major new group of rhabdoviruses.

Although the sigma viruses form a well-supported monophyletic clade, the relationships between the three viruses are uncertain. While the Bayesian analysis gives significant posterior support for relationships shown in figure 3, the more conservative maximum-likelihood bootstrapping does not support this topology (figure 3). As DMelSV is vertically transmitted, we were interested in whether the topology of the virus phylogeny differs from that of the host, which would indicate that the virus has switched hosts during its evolution rather than co-speciating with them. However, when we forced the topology of the sigma virus phylogeny to match the host phylogeny, there was no significant reduction in the likelihood of the tree (SH test: *p* = 0.173, difference in log likelihood=5.24). Therefore, we are unable to reject the hypothesis that the host and viral tree topologies are the same.

Our analysis produced a robust and well-supported phylogeny of the rhabdoviruses (figure 2). The rhabdoviruses contain two major clades, with the fish-infecting novirhabdoviruses forming a clade basal to all the other genera. In the other group, the arthropod-vectored plant viruses (cytorhabdoviruses and nucleorhabdoviruses) form a clade that is a sister group to the lyssaviruses, sigma viruses and the dimarhabdovirus supergroup. The dimarhabdovirus group (figure 3) contains the vesiculoviruses, the ephemeroviruses and some other viruses which are unassigned or have only tentatively been placed to this group (ICTVdB 2006). The sigma virus clade forms a sister group to all the other dimarhabdoviruses.

To assess whether our results are sensitive to the sequence alignment or method of phylogenetic reconstruction, we produced a total of six Bayesian trees and three maximum-likelihood trees (see §2). There was greater resolution in the Bayesian nucleotide trees with rate variation between codon positions; hence we presented these trees in the figures. However, when different methods of analysis were used, similar tree topologies were inferred. Furthermore, the conserved region alignment (electronic supplementary material, figure S1) and full-length sequence alignment (figure 2) lead to the same general conclusions. In addition to the uncertain relationships among the sigma viruses, there are other minor inconsistencies between the different trees. In the lyssavirus genus, depending on the method and alignment used, the branching order of the clade containing Arravan, Khujand and rabies viruses switched positions, possibly owing to the very short branch lengths in this group (Kuzmin *et al*. 2006). Also the branching order between taro vein chlorosis virus, Iranian maize mosaic and maize mosaic virus was sensitive to the method and alignment used.

## 4. Discussion

### (a) *Drosophila* sigma viruses

We have discovered two new rhabdoviruses in *D. affinis* and *D. obscura*, which together with DMelSV, brings the total number of insect-restricted rhabdoviruses to three. We sequenced the complete genomes of two of these viruses and partial genome of the third, and found that they form a major new clade on the rhabdovirus phylogeny (figure 2; see also Hogenhout *et al*. 2003; Kuzmin *et al*. 2006). This new clade does not fit into any existing genera as it is a sister group to the dimarhabdoviruses, which itself contains two genera. Additionally, the divergence between these viruses is greater than that seen within four of the six previously classified genera. We therefore suggest these three *Drosophila* viruses be regarded as a new genus.

As DMelSV, and probably the other sigma viruses, are vertically transmitted, it is interesting to ask whether the sigma viruses have co-speciated with their hosts or have moved horizontally between species during their evolution. If parasites have moved between hosts, this can result in incongruence between host and parasite phylogenies. However, all the three sigma viruses diverged from their common ancestor at a same time, and we are unable to tell whether or not the viral phylogeny matches the host phylogeny. Despite this, it seems likely that the viruses have switched between hosts owing to the length of branches on the tree. If these viruses had co-speciated with their hosts, we would expect the DAffSV and DObsSV to be much more closely related to one another than to DMelSV, given that *D. obscura* and *D. affinis* diverged from each other approximately 15–18 Myr and from *D. melanogaster* approximately 30–35 Myr (Gao *et al*. 2007). This is not the case, as the viruses all shared a common ancestor at a same time, suggesting that horizontal transfer has occurred. As these sigma viruses are all probably vertically transmitted, it is not clear how they could move between species. One possibility is that they can be vectored by the parasitic mites that feed on *Drosophila*. These mites have been shown to vector *Spiroplasma* bacteria (Jaenike *et al*. 2007) and are suspected to transfer transposable elements between species of *Drosophila* (Loreto *et al*. 2008). Furthermore, we have found DObsSV in mites removed from wild-caught flies (B. Longdon 2008, unpublished data), although we would highlight it is not known if the virus replicates in the mites or if they can transmit sigma horizontally.

It is possible that rhabdoviruses may be common parasites of insects. DAffSV was discovered in *D. affinis*, where there had been previous reports of CO_2_ sensitivity (Williamson 1961) and CO_2_ sensitivity has been described in 15 other species of *Drosophila* (Brun & Plus 1980). Therefore, our results suggest that many of these species may also be infected (although other viruses may cause flies to die in anoxic conditions, see §1 and Teninges *et al*. 1979). The second of these new viruses was found in *D. obscura* where CO_2_ sensitivity had not previously been reported. Given that we performed only a limited sampling of a few species, this also suggests that there may be many other insect rhabdoviruses waiting to be discovered.

Although the new sigma virus isolates are anciently divergent from other rhabdoviruses, their genomes are typical of the family. The genomes of DObsSV and DMelSV are similar, both containing six open reading frames, which correspond to the N-P-X-M-G-L genes (3′–5′). Based on sequence conservation and from the analysis of predicted proteins, five of the six genes are homologous to genes found in other rhabdoviruses and probably have similar functions. In contrast, the X genes in DObsSV and DMelSV share no detectable sequence similarity either to each other or to other rhabdovirus genes. Although both X genes encode proteins with a signal peptide and domains similar to viral RNA polymerases, their function remains a matter for speculation. Interestingly, the cytorhabdoviruses, the nucleorhabdoviruses and the wongabel and Flanders viruses all contain at least one gene between the P and M genes, some of which are similar sizes, raising the possibility that these genes may be orthologous to the X gene, but have diverged to such an extent that there is no detectable sequence similarity between them.

### (b) Rhabdovirus phylogeny

Our analysis has produced the most robustly supported phylogeny of the *Rhabdoviridae* to date, with the members of the various genera forming distinct, well-supported clades. By using conserved L gene sequence, we are able to root our trees using human parainfluenza virus 1, which allows us to examine the branching order at the base of the tree for the first time. Furthermore, in previous phylogenetic analyses the boundaries between genera in the dimarhabdovirus super group have been unclear (Bourhy *et al*. 2005). This has been resolved in our analyses, which has well-supported fine-scale resolution within this clade.

It has been suggested that the N gene should be used to obtain fine-scale resolution (Kuzmin *et al*. 2006). However, we have found that the more rapidly evolving regions of the L gene coupled to its large size (approx. 6 kb) provides a much greater phylogenetic resolution than the N gene even when looking at closely related viruses. Furthermore, using sequence from less-conserved regions such as the N gene can result in inaccurate sequence alignments, which in turn can result in an incorrect phylogeny (Ogden & Rosenberg 2006). This may explain why previous analyses have sometimes placed DMelSV in very different places in the rhabdoviruses tree (Hogenhout *et al*. 2003; Kuzmin *et al*. 2006, 2009). In addition, using the L gene has the benefit of allowing rapid detection of novel rhabdoviruses, by using a diagnostic PCR with conserved degenerate primers.

It is striking how viruses which infect similar hosts have a strong tendency to cluster together on the phylogeny, indicating that it is rare for rhabdoviruses to switch between distantly related hosts. Although it has been suggested that the ancestor of the *Rhabdoviridae* may have infected insects (Hogenhout *et al*. 2003), our results suggest that it may be premature to draw any conclusions on the origin of the group, as there appear to be two equally parsimonious models. Specifically, because the fish-infecting novirhabdoviruses are sister to all other groups, it is possible either that (i) the common ancestor of the rhabdovirus infected arthropods (insects or other crustaceans) and switched to fish on the lineage leading to the novirhabdoviruses, or (ii) the common ancestor infected fish and switched to arthropods on the lineage leading to the other six clades. Perhaps, more importantly, because there are likely to be many undiscovered rhabdoviruses in many different groups of hosts, any inferences about the ancestral ecology of this group would be extremely tentative at best. Nevertheless, it is likely that the ancestor of six of the seven major clades (all rhabdoviruses other than the novirhabdoviruses) infected arthropods, as these clades (with the exception of the lyssaviruses) include viruses which infect arthropods.

There have been a number of transitions between host taxa. There is evidence for three switches between aquatic and terrestrial habitats—one between the fish infecting novirhabdoviruses and the terrestrial viruses, and two within the dimarhabdoviruses—and a single transition to infect plants in the cytorhabdoviruses and nucleorhabdoviruses. In the clade containing the sigma viruses, dimarhabdoviruses and lyssaviruses, there has either been two events leading to these viruses gaining the ability to infect vertebrates, or one gain followed by a loss in the sigma virus clade. The incomplete sampling of rhabdoviruses means that there may be many more major host switches to be discovered.

In our phylogeny, the sigma viruses are a sister group to the dimarhabdoviruses, which suggests that the common ancestor of this group infected arthropods. Support for this argument comes from the ability of vesicular stomatitis virus to replicate in a range of insects, including sand flies, black flies, *Drosophila*, leafhoppers and moths (Tesh *et al*. 1972; Lastra & Esparza 1976; Rosen 1980; Mead *et al*. 2004). In addition, like the sigma virus, this virus can be transmitted transovarially in sandflies (Tesh *et al*. 1972). It is even possible that all the dimarhabdoviruses may infect arthropods, including the fish viruses that are usually assumed to be vertebrate-specific. For example, a virus 99 per cent identical to spring viraemia of carp has been found in penaeid shrimps (Johnson *et al*. 1999), we have found that EST libraries from fish lice contain rhabdovirus-like sequences (B. Longdon 2008, unpublished observation), and spring viraemia of carp can be vectored by sea lice in the laboratory (Ahne *et al*. 2002).

## 5. Conclusions

From a quick survey of *Drosophila* we have found two new viruses, which together with DMelSV form a major new clade in the *Rhabdoviridae*. It is possible that there is a great deal of diversity in this family yet to be discovered, and a more extensive survey for new rhabdoviruses may uncover viruses from a wide diversity host taxa and further our understanding of the relationships among the *Rhabdoviridae*.

## References

[RSPB20091472C1] Ahne W. Bjorklund H. V. Essbauer S. Fijan N. Kurath G. Winton J. R. 2002 Spring viremia of carp (SVC). Dis. Aquat. Organ. 52, 261–272 (doi:10.3354/dao052261) 12553453 10.3354/dao052261

[RSPB20091472C2] Ammar E. D. Tsai C. W. Whitfield A. E. Redinbaugh M. G. Hogenhout S. A. 2009 Cellular and molecular aspects of rhabdovirus interactions with insect and plant hosts. Ann. Rev. Entomol. 54, 447–468 (doi:10.1146/annurev.ento.54.110807.090454) 18793103 10.1146/annurev.ento.54.110807.090454

[RSPB20091472C3] Bendtsen J. D. Nielsen H. von Heijne G. Brunak S. 2004 Improved prediction of signal peptides: SignalP 3.0. J. Mol. Biol. 340, 783–795 15223320 10.1016/j.jmb.2004.05.028

[RSPB20091472C4] Berkalof A. Breglian J. Ohanessi A. 1965 Mise en evidence de virions dans des drosophiles infectees par le virus hereditaire sigma. Comptes Rendus Hebdomadaires des Seances de L'Academie des Sciences 260, 5956–5959 4953980

[RSPB20091472C5] Bjorklund H. V. Higman K. H. Kurath G. 1996 The glycoprotein genes and gene junctions of the fish rhabdoviruses spring viremia of carp virus and hirame rhabdovirus: analysis of relationships with other rhabdoviruses. Virus Res. 42, 65–80 (doi:10.1016/0168-1702(96)01300-7) 8806175 10.1016/0168-1702(96)01300-7

[RSPB20091472C6] Bourhy H. Cowley J. A. Larrous F. Holmes E. C. Walker P. J. 2005 Phylogenetic relationships among rhabdoviruses inferred using the L polymerase gene. J. Gen. Virol. 86, 2849–2858 (doi:10.1099/vir.0.81128-0) 16186241 10.1099/vir.0.81128-0

[RSPB20091472C7] Bras F. Teninges D. Dezelee S. 1994 Sequences of the N-gene and M-gene of the sigma-virus of *Drosophila* and evolutionary comparison. Virology 200, 189–199 (doi:10.1006/viro.1994.1177) 8128620 10.1006/viro.1994.1177

[RSPB20091472C8] Brun G. Plus N. 1980 The viruses of *Drosophila*. In The genetics and biology of Drosophila (eds Ashburner M. Wright T. R. F.), pp. 625–702 New York, NY: Academic Press

[RSPB20091472C9] Carpenter J. A. Obbard D. J. Maside X. Jiggins F. M. 2007 The recent spread of a vertically transmitted virus through populations of *Drosophila melanogaster*. Mol. Ecol. 16, 3947–3954 (doi:10.1111/j.1365-294X.2007.03460.x) 17725574 10.1111/j.1365-294X.2007.03460.x

[RSPB20091472C10] Contamine D. Gaumer S. 2008 Sigma rhabdoviruses. Encyclop. Virol. 5, 576–581

[RSPB20091472C11] Douady C. J. Delsuc F. Boucher Y. Doolittle W. F. Douzery E. J. P. 2003 Comparison of Bayesian and maximum likelihood bootstrap measures of phylogenetic reliability. Mol. Biol. Evol. 20, 248–254 (doi:10.1093/molbev/msg042) 12598692 10.1093/molbev/msg042

[RSPB20091472C12] Fu Z. F. 2005 Genetic comparison of the rhabdoviruses from animals and plants. World Rhabdoviruses 292, 1–24 (doi:10.1007/3-540-27485-5_1) 10.1007/3-540-27485-5_1 15981465

[RSPB20091472C13] Gao J. J. Watabe H. A. Aotsuka T. Pang J. F. Zhang Y. P. 2007 Molecular phylogeny of the *Drosophila obscura* species group, with emphasis on the Old World species. BMC Evol. Biol. 7, 87 17555574 10.1186/1471-2148-7-87 PMC1904182

[RSPB20091472C14] Gay P. 1978 *Drosophila* genes which intervene in multiplication of sigma virus. Mol. Gen. Genet. 159, 269–283 (doi:10.1007/BF00268263) 416336 10.1007/BF00268263

[RSPB20091472C15] Hogenhout S. A. Redinbaugh M. G. Ammar E. D. 2003 Plant and animal rhabdovirus host range: a bug's view. Trends Microbiol. 11, 264–271 (doi:10.1016/S0966-842X(03)00120-3) 12823943 10.1016/s0966-842x(03)00120-3

[RSPB20091472C16] Huelsenbeck J. P. Ronquist F. 2001 MrBayes: Bayesian inference of phylogenetic trees. Bioinformatics 17, 754–755 (doi:10.1093/bioinformatics/17.8.754) 11524383 10.1093/bioinformatics/17.8.754

[RSPB20091472C17] Huszar T. Imler J. L. 2008 *Drosophila* viruses and the study of antiviral host-defense. Adv. Virus Res. 72, 227–265 (doi:10.1016/S0065-3527(08)00406-5) 19081493 10.1016/S0065-3527(08)00406-5

[RSPB20091472C18] ICTVdB 2006 Rhabdoviridae. ICTVdB: The Universal Virus Database, version 3 (ed. Büchen-Osmond C.). New York, NY: Columbia University

[RSPB20091472C19] Jaenike J. Polak M. Fiskin A. Helou M. Minhas M. 2007 Interspecific transmission of endosymbiotic *Spiroplasma* by mites. Biol. Lett. 3, 23–25 (doi:10.1098/rsbl.2006.0577) 17443956 10.1098/rsbl.2006.0577 PMC2373825

[RSPB20091472C20] Johnson M. C. Maxwell J. M. Loh P. C. Leong J. A. C. 1999 Molecular characterization of the glycoproteins from two warm water rhabdoviruses: snakehead rhabdovirus (SHRV) and rhabdovirus of penaeid shrimp (RPS)/spring viremia of carp virus (SVCV). Virus Res. 64, 95–106 (doi:10.1016/S0168-1702(99)00071-4) 10518707 10.1016/s0168-1702(99)00071-4

[RSPB20091472C21] Kelley L. Sternberg M. 2009 Protein structure prediction on the web: a case study using the Phyre server. Nat. Protocols 4, 363–471 (doi:10.1038/nprot.2009.2) 10.1038/nprot.2009.2 19247286

[RSPB20091472C22] Kuzmin I. V. Hughes G. J. Rupprecht C. E. 2006 Phylogenetic relationships of seven previously unclassified viruses within the family Rhabdoviridae using partial nucleoprotein gene sequences. J. Gen. Virol. 87, 2323–2331 (doi:10.1099/vir.0.81879-0) 16847128 10.1099/vir.0.81879-0

[RSPB20091472C23] Kuzmin I. V. Novella I. S. Dietzgen R. G. Padhi A. Rupprecht C. E. 2009 The rhabdoviruses: biodiversity, phylogenetics, and evolution. Infect., Genet. Evol. 9, 541–553 (doi:10.1016/j.meegid.2009.02.005) 19460320 10.1016/j.meegid.2009.02.005

[RSPB20091472C24] Landesdevauchelle C. Bras F. Dezelee S. Teninges D. 1995 Gene 2 of the sigma rhabdovirus genome encodes the p-protein, and gene-3 encodes a protein related to the reverse-transcriptase of retroelements. Virology 213, 300–312 (doi:10.1006/viro.1995.0003) 7491755 10.1006/viro.1995.0003

[RSPB20091472C25] Lastra J. Esparza J. 1976 Multipication of vesicular stomatitis virus in the leafhopper *Peregrinus maidis* (Ashm.), a vector of a plant rhabdovirus. J. Gen. Virol. 32, 139–142 (doi:10.1099/0022-1317-32-1-139) 182911 10.1099/0022-1317-32-1-139

[RSPB20091472C26] L'Heritier P. H. Teissier G. 1937 Une anomalie physiologique héréditaire chez la Drosophile. C.R. Acad. Sci. Paris 231, 192–194

[RSPB20091472C27] Loreto E. L. S. Carareto C. M. A. Capy P. 2008 Revisiting horizontal transfer of transposable elements in *Drosophila*. Heredity 100, 545–554 (doi:10.1038/sj.hdy.6801094) 18431403 10.1038/sj.hdy.6801094

[RSPB20091472C28] Mead D. G. Howerth E. W. Murphy M. D. Gray E. W. Noblet R. Stallknecht D. E. 2004 Black fly involvement in the epidemic transmission of vesicular stomatitis New Jersey virus (Rhabdoviridae: Vesiculovirus). Vector-Borne Zoonot. Dis. 4, 351–359 (doi:10.1089/vbz.2004.4.351) 10.1089/vbz.2004.4.351 15671739

[RSPB20091472C29] Ogden T. H. Rosenberg M. S. 2006 Multiple sequence alignment accuracy and phylogenetic inference. Syst. Biol. 55, 314–328 (doi:10.1080/10635150500541730) 16611602 10.1080/10635150500541730

[RSPB20091472C30] Poch O. Sauvaget I. Delaure M. Tordo N. 1989 Identification of four conserved motifs among the RDRP encoding elements. EMBO J. 8, 3867–3874 2555175 10.1002/j.1460-2075.1989.tb08565.x PMC402075

[RSPB20091472C31] Poch O. Blumberg B. M. Bougueleret L. Tordo N. 1990 Sequence comparison of 5 polymerases (L-proteins) of unsegmented negative-strand RNA viruses: theoretical assignment of functional domains. J. Gen. Virol. 71, 1153–1162 (doi:10.1099/0022-1317-71-5-1153) 2161049 10.1099/0022-1317-71-5-1153

[RSPB20091472C32] Posada D. Crandall K. A. 1998 MODELTEST: testing the model of DNA substitution. Bioinformatics 14, 817–818 (doi:10.1093/bioinformatics/14.9.817) 9918953 10.1093/bioinformatics/14.9.817

[RSPB20091472C33] Rosen L. 1980 Carbon-dioxide sensitivity in mosquitos infected with sigma, vesicular stomatitis, and other rhabdoviruses. Science 207, 989–991 (doi:10.1126/science.6101512) 6101512 10.1126/science.6101512

[RSPB20091472C34] Shimodaira H. Hasegawa M. 1999 Multiple comparisons of log-likelihoods with applications to phylogenetic inference. Mol. Biol. Evol. 16, 1114–1116

[RSPB20091472C35] Shroyer D. A. Rosen L. 1983 Extrachromosomal-inheritance of carbon-dioxide sensitivity in the mosquito *Culex* *quinquefasciatus*. Genetics 104, 649–659 6413297 10.1093/genetics/104.4.649 PMC1202131

[RSPB20091472C36] Swofford D. L. 1993 PAUP*. Phylogenetic analysis using parsimony (*and other methods). Version 4 Sunderland, MA: Sunderland Associates

[RSPB20091472C37] Sylvester E. Richardson J. 1992 Aphid-borne rhabdoviruses-relationship with their aphid vectors. In Advances disease vector research, vol. 9 (ed. Harris K. F.), pp. 313–341 New York, NY: Springer-Verlag

[RSPB20091472C38] Teninges D. 1968 Demonstration of sigma viruses in the cells of the stabilized male germinal line of *Drosophila*. Arch Gesamte Virusforsch 23, 378–387 (doi:10.1007/BF01242133) 5680979

[RSPB20091472C39] Teninges D. Ohanessian A. Richardmolard C. Contamine D. 1979 Isolation and biological properties of *Drosophila* X-virus. J. Gen. Virol. 42, 241–254 (doi:10.1099/0022-1317-42-2-241)

[RSPB20091472C40] Teninges D. Bras F. Dezelee S. 1993 Genome organization of the sigma rhabdovirus—6 genes and a gene overlap. Virology 193, 1018–1023 (doi:10.1006/viro.1993.1219) 8384742 10.1006/viro.1993.1219

[RSPB20091472C41] Tesh R. B. Byron N. C. Johnson K. M. 1972 Vesicular stomatitis virus (Indiana serotype): transovarial transmission by phlebotomine sandflies. Science 175, 1477–1479 (doi:10.1126/science.175.4029.1477) 4335268 10.1126/science.175.4029.1477

[RSPB20091472C42] Williamson D. L. 1959 Carbon dioxide sensitivity in *Drosophila affinis* and *Drosophila athabasca*. PhD thesis, University of Nebraska 10.1093/genetics/46.8.1053 PMC1210249 13785540

[RSPB20091472C43] Williamson D. 1961 Carbon dioxide sensitivity in *Drosophila affinis* and *Drosophila athabasca*. Genetics 46, 1053–105& 13785540 10.1093/genetics/46.8.1053 PMC1210249

[RSPB20091472C44] Zdobnov E. M. Apweiler R. 2001 InterProScan: an integration platform for the signature-recognition methods in InterPro. Bioinformatics 17, 847–848 (doi:10.1093/bioinformatics/17.9.847) 11590104 10.1093/bioinformatics/17.9.847

